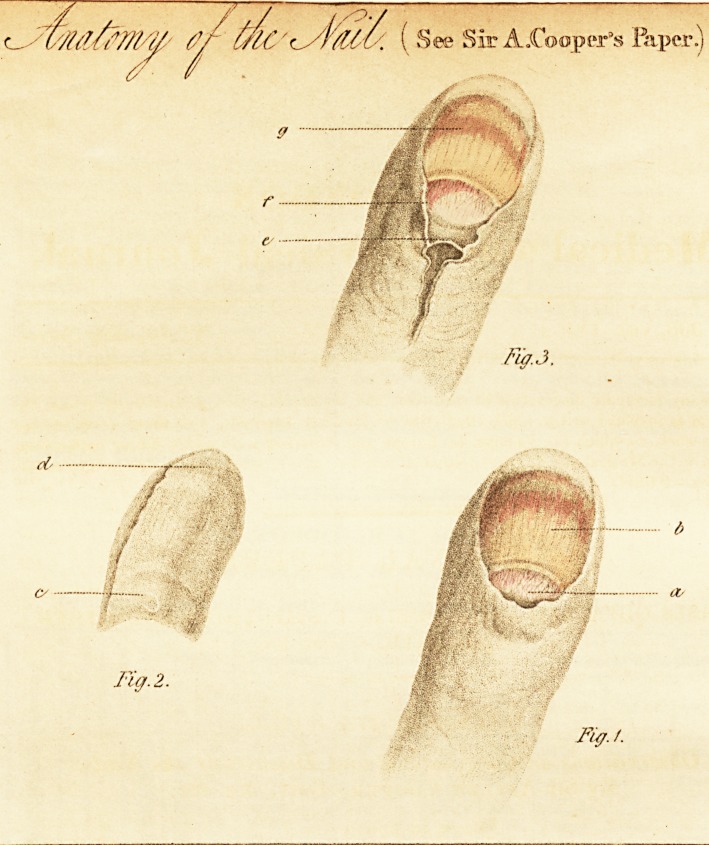# Observations on the Anatomy and Diseases of the Nails

**Published:** 1827-04

**Authors:** Astley Cooper

**Affiliations:** Bart, &c. &c.


Fig. 3.

Fig. 2.

Fig. 1.

### Catarrho-Rheumatic Ophthalmia. (See Prof.r Mackenzie's Paper, Case VIII.)

Fig. 1.

Fig. 2.

H. Adlard Sc.

### THE LONDON
### Medical and Physical Journal.

Nº 338, VOL LVII.] APRIL, 1827. [Nº 10, *New Series*.

For many fortunate discoveries in medicine, and for the detection of numerous errors, the world is indebted to the rapid circulation of Monthly Journals; and there never existed any work, to which the Faculty, in Europe and America, were under deeper obligations, than to the Medical and Physical Journal of London, now forming a long, but an invaluable, series.—RUSH.

### ORIGINAL PAPERS,
AND
### CASES OBTAINED FROM PUBLIC INSTITUTIONS AND OTHER AUTHENTIC SOURCES.

### DISEASE OF THE NAILS.

*Observations on the Anatomy and Diseases of the Nails.*
By Sir ASTLEY COOPER, Bart, &c. &c.

[WITH AN ENGRAVING.]

IN reply to your inquiries of what I have so long taught upon the diseases of the nails, and the structure by which they are produced, I with pleasure send you the following account; although that part which relates to their diseases you will find in my published lectures, and the anatomical description has been given in my anatomical course.

*Of the Nail.*—When this part is separated by putrifaction, and its internal surface is examined, it is found to be divided into three parts: viz.—1st, a hollow and nearly smooth white surface, at its root; 2dly, a hollow white laminated surface, in its middle; 3dly, a hollow, brownish, and less distinctly laminated portion, near its extremity.

*Of the Ungual Surface beneath the Nail.*—This is divided into two parts. Opposite to the hollow at the root of the nail is placed a highly vascular and villous surface, which I call the ungual gland, and the portion of the nail over this surface is thinner than the rest. Beyond this secreting surface appear a number of laminæ, like the under part of the mushroom, which are parallel with those placed in the inner part of the nail, and which pass in the direction of the axis of the finger. The parts of the nail usually cut project beyond these laminæ.

The ungual gland is a very vascular surface, and its use is to secrete the nail, which proceeds from it between the laminæ placed before it; so that the nail grows from its root, as may be easily seen by cutting a notch there, which grows gradually out in about three months, advancing until it reaches the extremity of the nail. The growth of a new nail also illustrates this position.

The laminæ situated anteriorly to the secreting surface, and upon the third phalanx of the finger, are highly vascular, as far as the adhesion of the nail extends; but beyond this the cuticle of the end of the finger turns in to unite itself to the laminæ. Their vessels are arteries and veins, the latter of which form a plexus, with very frequent communications. The nail adheres to the finger by the cuticle, and it therefore separates by putrefaction and boiling: it also adheres at its root to the secreting surface which produces it; and, above all, it adheres by its laminæ being received between the living laminæ beneath. Opposite to the root of the nail, the cutis and cuticle are double, and turn inwards; so that a considerable portion of the nail is covered by the common integuments. The cuticle unites to the nail; the cutis passes under it, to produce the secreting surface and laminæ,—it is vascular and villous, that it may secrete the nail; vascular and laminated, in order to produce the adhesion of the nail to the skin.

*On the diseased Growth of the Nail.*—The nail sometimes grows broader than it ought, and it then produces ulceration by the pressure of its edge, which is followed by an irritable and fungous granulation. As this state arises from the breadth of the nail, and its consequent pressure, it sometimes continues for months, or even for years; yet it will yield to proper treatment in two or three weeks. The common mode of relief consists in cutting a notch in the centre of the nail; in scraping its extremity thin; in putting it frequently in warm water, and in putting a piece of lint under its projecting edge: but this mode often fails in producing a cure, and frequently is only a temporary relief. In obstinate and difficult cases of this unnatural growth of the nail, I have, for thirty-five years, recommended and practised the plan of cutting away the edge of the nail with scissors, from its extremity to its root; by which a cure is often produced in a few days, and in the worst cases in two or three weeks. A poultice only is afterwards required.

*Of Disease in the Ungual Gland.*—In diseased states of the constitution, the secreting surface which produces the nail gets into a morbid state, and, instead of a healthy nail being formed, it throws out one which is black, everted, unadherent, and which so irritates the vascular surfaces as to produce an irritable, sloughing, and very painful sore, which renders the patient lame, so as to prevent his gaining his daily bread. As this is a constitutional as well as local disease, it becomes necessary to employ constitutional and local means of treatment. My usual plan is to give a grain of Calomel, with a grain of Opium, night and morning, with the Decoctum Sarsaparillæ Compositum; and to apply the Liquor Calcis ℥iv. with Calomel ʒj. by means of lint with oiled silk over it. This plan often succeeds; and, if it does not, it destroys the predisposition to the disease.

After giving these constitutional remedies, if the sore does not heal, I have sometimes applied a blister to bring off the nail, and alter the action of the ulcer. But in hospital practice, where persons are anxious to return to their labour, and to have their disease quickly and effectually removed, I have always dissected away the secreting surface which produces the nail, and prevented the possibility of a recurrence of the disease.

The plate which accompanies this will serve to illustrate the anatomy of the parts.

*Description of the Plate.*

Fig. 1.—*a*, Secreting part,
*b*, Laminæ.

Fig. 2.—*c*, Hollow for the gland, or secreting part,
*d*, Projecting part.

Fig. 3.—*e*, Skin turned under the nail,
*f*, The secreting surface,
*g*, Laminæ.

The skin turned under the nail becomes, by change of structure, the secreting surface.

*New-street, Spring Gardens; February 7th.*

## Figures and Tables

**Fig. 3. Fig. 2. Fig. 1. f1:**